# Programmed cell death disrupts inflammatory tumor microenvironment (TME) and promotes glioblastoma evolution

**DOI:** 10.1186/s12964-024-01602-0

**Published:** 2024-06-18

**Authors:** Tingyu Liang, Lingui Gu, Xiaoman Kang, Junlin Li, Yixuan Song, Yu Wang, Wenbin Ma

**Affiliations:** 1 Department of Neurosurgery, Center for Malignant Brain Tumors, National Glioma MDT Alliance, Peking Union Medical College Hospital, Chinese Academy of Medical Sciences and Peking Union Medical College, Beijing, 100730 China; 2 Eight-year Medical Doctor Program, Chinese Academy of Medical Sciences and Peking Union Medical College, Beijing, 100730 China; 3 ‘4+4’ Medical Doctor Program, Chinese Academy of Medical Sciences and Peking Union Medical College, Beijing, 100730 China; 4 Department of Neurosurgery, Peking Union Medical College Hospital, Chinese Academy of Medical Sciences and Peking Union Medical College, Beijing, 100730 China

**Keywords:** GBM, PCD, Inflammatory TME, Treatment, Tumor evolution

## Abstract

Glioblastoma (GBM) is the most common malignant brain tumor and has a dismal prognosis even under the current first-line treatment, with a 5-year survival rate less than 7%. Therefore, it is important to understand the mechanism of treatment resistance and develop new anti-tumor strategies. Induction of programmed cell death (PCD) has become a promising anti-tumor strategy, but its effectiveness in treating GBM remains controversial. On the one hand, PCD triggers tumor cell death and then release mediators to draw in immune cells, creating a pro-inflammatory tumor microenvironment (TME). One the other hand, mounting evidence suggests that PCD and inflammatory TME will force tumor cells to evolve under survival stress, leading to tumor recurrence. The purpose of this review is to summarize the role of PCD and inflammatory TME in the tumor evolution of GBM and promising methods to overcome tumor evolution.

## Background

Glioblastoma (GBM), the most common type of malignant glioma and primary brain tumor, is still one of the most fatal tumors, with a 5-year survival rate of 6.9% [1, 2]. Due to its late diagnosis, aggressive infiltration, and higher inter- and intra-tumoral genetic heterogeneity, GBM has limited treatment choices [3]. Tumor evolution, defined by genetic and biological adaptations, is a crucial approach for tumor cells to cope with the complicated living environment, especially when tumor cells are under the survival stress imposed by various anti-tumor treatment. It is worth noting that selective pressure might force tumor cells to evolve along different pathways. Using both bulk and single-cell data, many studies on GBM have shown different evolution models of the GBM genome, including gradualism and punctuated evolution [4, 5]. Therefore, tumor evolution can result in the heterogeneity and therapeutic resistance in GBM [6]. For instance, the proteomic landscape of 134 primary GBM (pGBM) and recurrent GBM (rGBM) samples, including 40 paired pGBM – rGBM ones, reveals that tumor recurrence is associated with post-treatment tumor evolution, by activating conventional tumor-promoting pathways such as epithelial-mesenchymal transition (EMT) [7]. Another study by Piao et al. shows that anti-vascular endothelial growth factor treatment can induce hypoxic tumor microenvironment (TME) and stimulate myeloid cell infiltration, as well as tumor mesenchymal transition, promoting tumor progression [8]. Therefore, confirming the drivers of tumor evolution is essential for understanding how GBM tumor cells evade cancer treatment.

Programmed cell death (PCD) eliminates unwanted cells to maintain the physiological homeostasis through several distinctive pathways, with the major types including apoptosis, autophagy, ferroptosis and pyroptosis [9]. Apoptosis, the classical and extensively studied type of PCD, is characterized by release of cytochrome C from injured mitochondria [10]. Apoptotic cell death is regulated by the delicate balance between pro-apoptotic and anti-apoptotic proteins to activate downstream caspases, initiating the well-characterized process including cell shrinkage, chromatin condensation and DNA fragmentation [11]. Autophagy (PCD type II), on the contrary, is critical for sustaining cell viability under stress rather than triggering cell death as other forms of PCD [12]. With the administration of chemotherapy or other anti-tumor treatment, PI3K/Akt/mTOR signaling is suppressed, forcing GBM cells to activate autophagy as a protective response [12–14]. Autophagy is also enhanced when Wnt signaling is activated to regulate cell proliferation and migration [12]. Ferroptosis, a relatively newly discovered form of PCD, occurs due to the intracellular accumulation of iron and reactive oxygen species (ROS) when poly unsaturated fatty acids undergo lipid peroxidation, thus disrupting the intracellular redox balance [12]. Lastly, pyroptosis, another recently discovered form of PCD, is involved in immune activation, contrasting with the immunosuppressive nature of apoptosis [12]. It is marked by cell swelling and subsequent membrane rupture, releasing intracellular components to attract inflammatory cells and activate immune responses [10, 12]. Many studies have shown the powerful anti-tumor properties of PCD in GBM. For instance, Apatinib, a tyrosine kinase inhibitor, can induce ferroptosis in glioblastoma cell lines and consequently inhibit tumor proliferation [15]. Furthermore, another antineoplastic therapy, Roxadustat, amplifies hypoxia-inducible factor (HIF) signaling to stimulate ferroptosis and suppress the growth of chemoresistant GBM cells [16]. Zhibo Liu and colleagues developed a biorthogonal system delivering gasdermin to tumor cells, which suggests gasdermin-induced pyroptosis may provoke robust anti-tumor immunity and improve the effectiveness of anti-PD-1 therapy in pan-cancer [17]. However, as more evidence uncovers the therapeutic effects of PCD, the role of PCD in tumor evolution, particularly its interaction with TME, starts to draw renewed attention in the scientific community. For example, tumor cells undergoing PCD secrete cytokines to prompt the immune cell infiltration and thus reshape the TME [18–20]. Consequently, the reshaped TME can transform the infiltrated immune cells into immune-suppressive types to support tumor development. Additionally, local immune cells in the TME can also undergo PCD themselves, leading to intratumoral immune suppression and tumor progression [21].

As mentioned above, the relationship between TME and tumor progression is intriguing and PCD can function as a double-edged sword in glioblastoma development. This article aims to summarize the contribution of PCD in the tumorigenic TME and subsequently tumor evolution in GBM. Furthermore, we summarize the dual function of PCD in TME formation, providing the basis of designing innovative therapeutic strategies to overcome treatment resistance in glioblastoma.

### PCD supports the cancer-immunity cycle and reshapes inflammatory TME

**Fig. 1:**
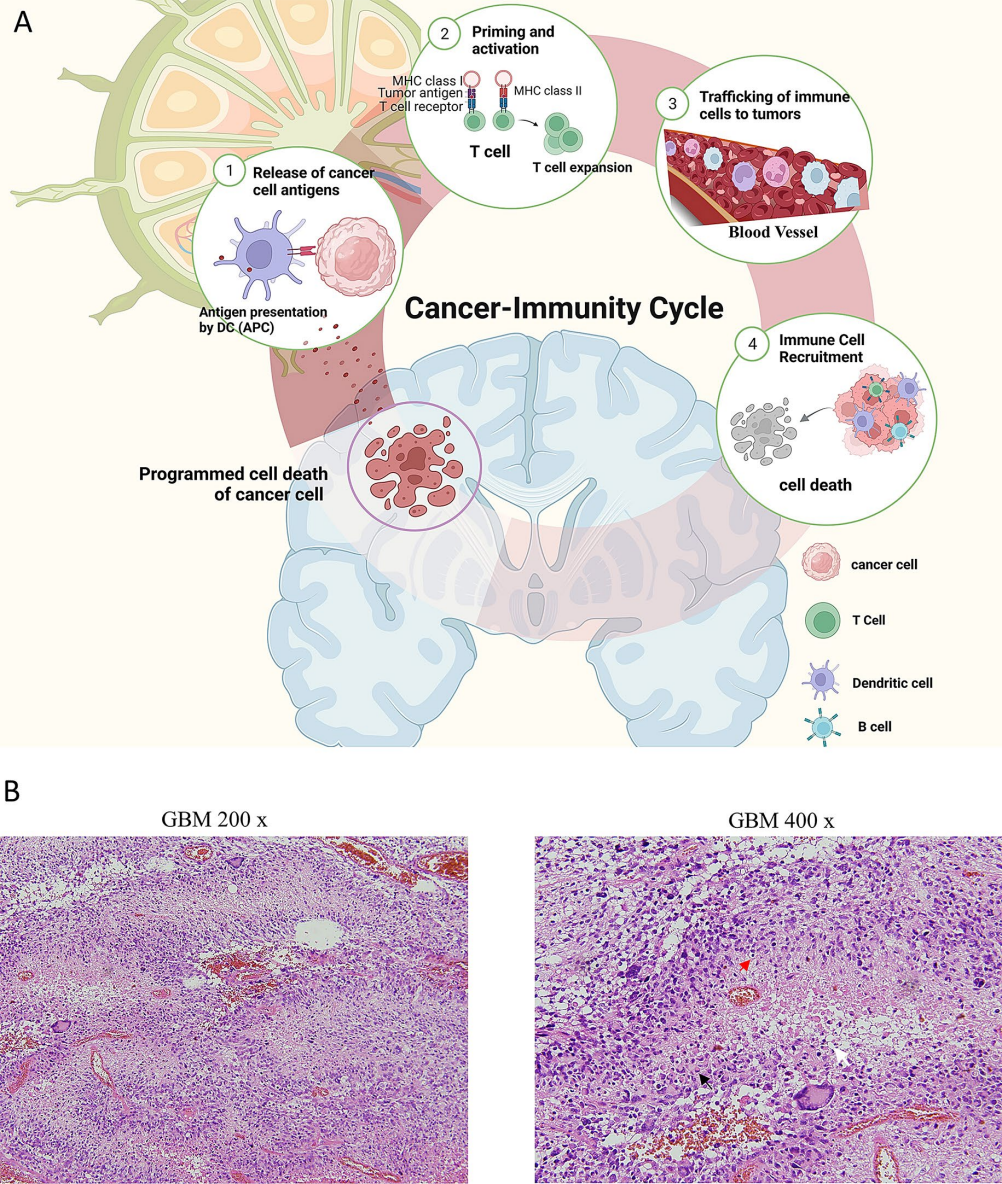
The overview of the cancer-immunity cycle. **A**: PCD can support and sustain the cancer-immunity cycle. PCD can induce immune system to recognize destroyed tumor cells, and recruit numerous immune cells into TME. The infiltrative immune cells can communicate with tumor cells. **B**: H&E-staining of GBM patient presents numerous lymphocytes (red arrow) and macrophages (black arrow) infiltration around necrotic region (white arrow)

PCD not only eliminates tumor cells directly through various types of cell death, but also allows GBM cells to release tumor antigens that will drain into cervical lymph nodes, provoking immunological responses. Afterwards, antigen-presenting cells (APCs) present the tumor antigens to and activate effector immune cells. These activated effector cells will then be trafficked to the brain tumor and initiate immunological destruction of tumor cells upon recognition of tumor antigens. As a result of immune infiltration, more tumor cells will undergo PCD and release increasing amount of tumor antigens, completing the cancer-immunity cycle (Fig. 1A). This can be illustrated by an H&E staining of the tumor tissue from a GBM patient, showing abundant immune cell infiltration, including lymphocytes and macrophages, around the central necrotic region (Fig. 1B). In recent years, many treatments for GBM have been shown to involve PCD as their anti-tumor actions. As one of the few treatments for GBM that have demonstrated substantial survival benefit in clinical trials, tumor treating fields (TTFields) can activate stimulator of interferon genes (STING) and absent in melanoma 2 (AIM2) inflammasomes to trigger pyroptosis in tumor cells, which will then release tumor antigens and recruit dendritic cell (DCs) as well as tumor-specific cytotoxic T lymphocytes (CTLs), thus converting the TME into a pro-inflammatory environment [20]. Unexpectedly, immune recruitment and infiltration in GBM are not necessarily associated with improved prognosis. In fact, PCD and immune infiltration might be predictive of worse clinical outcome as shown in bioinformatics analysis, in vitro and in vivo studies [22–25]. The seemingly contradictory evidence piques our curiosity in the exact role of PCD in GBM.

### Good-to-bad inflammatory microenvironment caused by PCD

Ever since the nineteenth century, there is ongoing debate about the possible link between inflammation and malignancies [26]. Early activation of acute inflammatory response can boost cytotoxic lymphocyte responses and elicit immune-mediated cancer cell death [27]. However, as the evidence accumulates, inflammation can be either pro-tumor or anti-tumor, depending on whether its presence is acute or persistent.

#### Acute inflammation caused by PCD kills the tumor

As previously discussed, the anti-tumor immune response triggered by PCD is comprised of pyroptosis, ferroptosis, and autophagy, all of which induce tumor cell death and then causes acute inflammation [28, 29]. Ferroptosis is a subtype of PCD characterized by iron-dependent lipid peroxidation [30]. Ferroptosis primarily limit glioblastoma cell survival through ROS generation to activate acute inflammation and enhance tumor cell apoptosis [31]. Its effectiveness in tumor suppression is exemplified by the fact that numerous cancer therapies act via ferroptosis, including preclinical medications (Erastin, RSL3), approved medications (sorafenib, sulfasalazine, statins, artemisinin) and radiation therapy [32, 33]. Pyroptosis, another subtype of PCD, is a newly discovered controlled cell death. Similar to ferroptosis, it also promotes cancer cell death and the subsequent release of inflammatory molecules, eliciting robust cytotoxic lymphocyte responses to prevent tumor progression [34, 35].

#### Long-term chronic inflammation modulates the TME

Although PCD-based immune-stimulating therapies are meant to detect and kill tumor cells, the anti-tumor effect of PCD is debatable in the actual clinical setting. GBM patients with enhanced ferroptosis have a higher risk of developing the disease, having a worse prognosis, and experiencing worse immunosuppression [36]. Clinical data also show that glioma patients expressing high level of pyroptosis-related genes(PRGs) have worse outcomes and are at greater risk of metastasis [37]. Moreover, compared to low-grade gliomas, high-grade gliomas have higher expression of PRGs probably associated with their malignant progression [38].

**Fig. 2:**
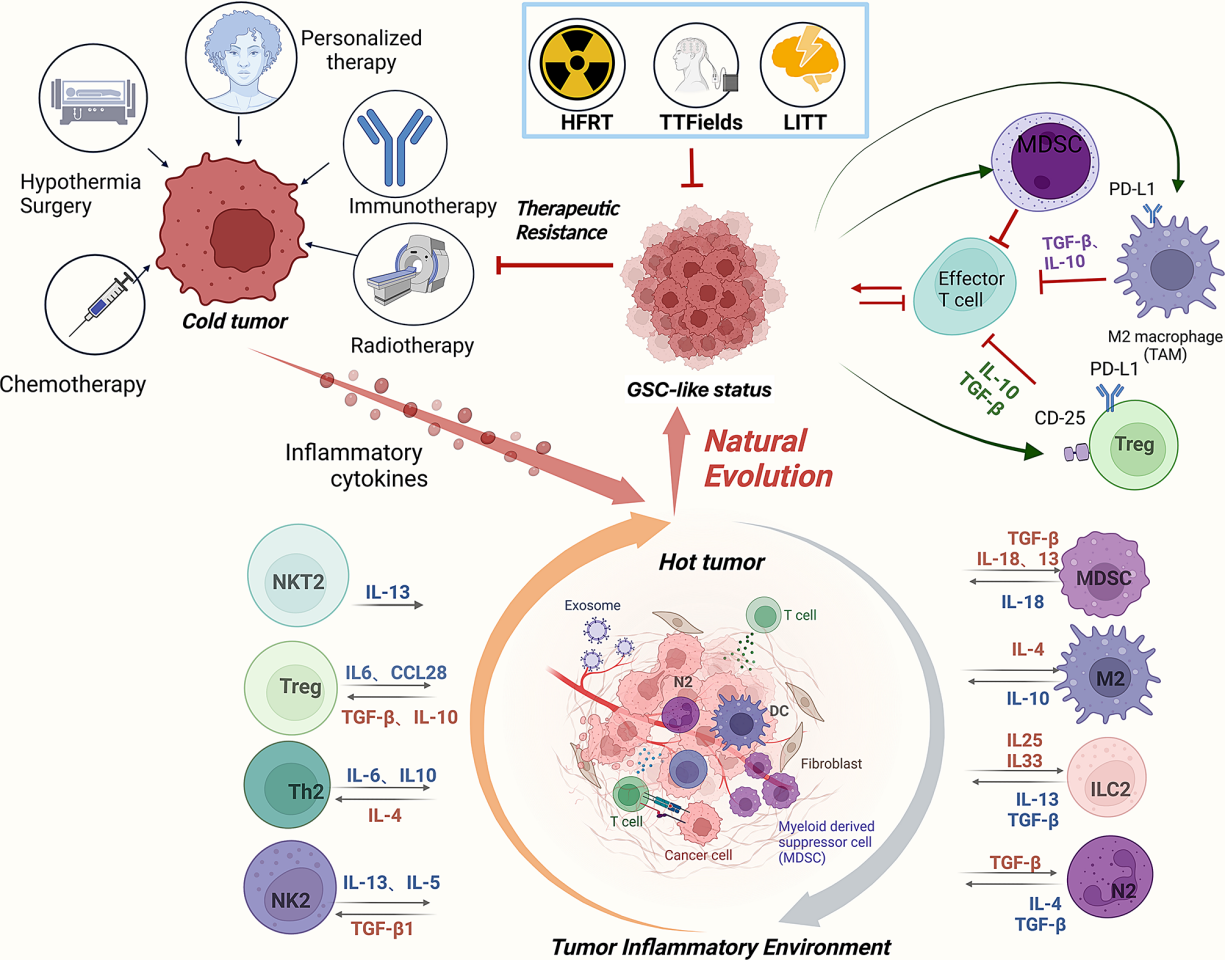
Treatments cause PCD and inflammatory TME to promote tumor evolution. PCD-based therapies such as chemotherapy, immune checkpoint inhibition and personalized immunotherapy can induce cancer cell death, exposing intracellular contents as immunogenic signals and transforming immunologically “cold tumor” into “hot tumor” with inflammatory TME. However, prolonged PCD may lead to functional exhaustion among infiltrating immune cells due to a sustained influx of inflammatory cytokines, resulting in the emergence of immune-suppressive cell types like N2 neutrophils, regulatory T cells (Treg), and the M2 subtype of tumor-associated macrophages (TAM). Under the selection pressure of chronic PCD and the resultant enduring inflammatory TME, tumor cells will adapt to the harsh environment through natural selection. The epithelial-mesenchymal transition (EMT) of GBM cells in the inflammatory TME leads to glioma stem cell (GSC) accumulation, driving tumor evolution and treatment resistance. Therefore, indiscriminate treatments, including TTFields, LITT and HFRT, can kill tumor cells intensively and inhibit tumor evolution. (ILC2: type 2 innate lymphoid cells; MDSC: myeloid-derived suppressor cells; NK2: type 2 natural killer cells; NKT2: type 2 natural killer T cells; Th2: type 2 T helper cells; Treg: regulatory T cells)

The reason behind PCD’s inadequacy in the clinical setting lies in the duration of immune stimulation. While preclinical studies prove that the short-term proinflammatory and immune-stimulating effect of PCD can suppress tumor proliferation, persisting proinflammatory immune response may alter the immunological microenvironment to favor tumor growth. Firstly, PCD induced inflammation drives M2 polarization of the infiltrative macrophages, a pro-tumor phenotype of macrophages. As demonstrated by Dai et al., the oxidative stress caused by ferroptosis in tumor cells will trigger the release of oncogenic KRAS protein, which is then taken up by local tumor-associated macrophages, causing them to switch to an M2 phenotype [39]. Zheng et al. develop a pyroptosis-related gene based prognostic index (PRGPI) and discover that patients with high PRGPI exhibit an extensively immune-suppressed TME, specifically higher infiltration of M2-type macrophages, lower infiltration of CD8 T cells and activated NK cells, and higher expression of immune checkpoints [38]. In addition to macrophages, ferroptosis also inhibit CD36^+^ T cells from releasing cytotoxic cytokines and tune down T cell antitumor activity, especially when paired with anti-PD-1 [40]. Inflammatory factors such as IL-18 may stimulate Th2 responses and angiogenesis, leading to enhanced tumor migration and invasion, and IL-1β can attract monocytes in the TME and operate as a master cytokine in cancer growth, as well as dampen T cell responses in the TME [41–44]. Furthermore, IL-6 can influence other cells within the TME to create a favorable growing environment for tumor cells, allowing for easier angiogenesis and tumor escape from immune surveillance [45]. Glioblastoma-derived IL-33 also promotes tumor growth by orchestrating an inflammatory TME [46]. Studies in many different tumors have shown that human regulatory B (Breg) cells secrete many cytokines (including IL-6, IL-10, IL-35, and TGF-β) to support the expansion of regulatory T cells (Tregs), tumor-associated macrophages (TAMs), and myeloid-derived suppressor cells (MDSCs) around the tumor bed, driving these cells toward immune-suppressive phenotypes to enhance the tumor-promoting microenvironment [47]. The prolonged immune stimulation brought on by PCD and inflammatory cytokines reshapes the TME into an immunosuppressive one and help tumor cells evade host immune surveillance. The long-term effect of PCD on promoting the development of an immunosuppressive milieu underscores the necessity to harness the tumor-killing effect of acute inflammation while impede the transformation into immunosuppressive TME for effective cancer treatment (Fig. 2).

### Tumor cells evolve to adapt to the PCD and inflammatory TME

The TME can influence tumor sensitivity to treatment by facilitating immune evasion and cancer progression [48]. PCD-induced proinflammatory TME is an independent predictor of poor prognosis in glioma patients and there is mounting evidence that the TME of GBM, the most malignant type of gliomas, has higher rates of immune cell infiltration and programmed cell death (PCD) scores compared to lower grade gliomas [49]. As the hallmark of malignant transformation, tumor evolution might be the reason behind the contradicting effect of PCD on the TME (Fig. 2). Under the pressure of tumor elimination, tumor cells will experience genomic alterations to adapt to the harsh living environment. For instance, PD-1/PD-L1 blockade can increase the amount of IFN-γ secreted by CD8^+^ T cells, which in turn trigger tumor cells to upregulate the Wnt/β-catenin signaling and accelerate the progression of malignant cancers [50]. Similarly, using in vivo CRISPR screens in the GBM mouse model, Stephen J. Elledge et al. discover that tumor cells only evolve to downregulate genes in antigen presenting pathway and upregulate inhibitory immune checkpoint genes in immunocompetent mice, but not in immunocompromised mice [51]. This study suggests that the survival pressure imposed by the pro-inflammatory immunity in TME will select and expand tumor cell clones with the capability of evading adaptive immune surveillance, thus provoking tumor evolution [51]. Furthermore, tumor destruction early on at the course of disease will recruit neutrophils and subsequently cause neutrophil-mediated ferroptosis in GBM cells, forming a positive feedback cycle [21]. However, this PCD-amplified immune infiltration educates neutrophils to deliver myeloperoxidase into GBM cells and as a result, tumor cells evolve by increasing the expression of mesenchymal-transition related genes [21]. Meanwhile, overexpression of ferroptosis-inhibiting molecules, such as glutathione peroxidase 4 (GPX4) which is an essential phospholipid peroxidase, can significantly alleviate PCD-mediated necrosis and diminish tumor aggressiveness [21]. All in all, tumor cells interact with the TME to drive malignant progression through evolution.

### PCD-induced inflammatory TME drives tumor evolution by facilitating EMT transformation and glioma stem cells (GSCs) accumulation

GSCs, a small and uncommon subset of glioma tissues, are characterized by their self-renewal and multi-lineage differentiation abilities and can be identified by their unique markers, such as CD133, CD44, VIM, and N-cadherin [52]. The tumor-initiating characteristics of GSCs lead to intratumoral heterogeneity, immunosuppressive TME, and treatment resistance [52]. Notably, GSCs may be responsible for tumor evolution and further reinforce the treatment resistance brought about by PCD. A growing body of research suggests that inflammatory TME and GSCs have a mutually beneficial relationship, implying that inflammatory TME might cause GSC conversion and tumor progression. For example, PCD can produce IFN-γ, a classical proinflammatory cytokine, to reshape the TME. The proinflammatory TME will then prompt GSCs to enter an immunological-insensitive state via the IDO-kynurenine-AHR pathway, resulting in chemotherapy resistance [53]. In addition to promote GSC accumulation through EMT signaling activation, the IFN-γ release upon PCD induction can upregulate the expression of immune checkpoints, such as PD-L1 in cancers, thus worsening the immune suppression in PCD-reshaped TME [54]. Likewise, other pro-inflammatory cytokines such as IL-6, IL-1, and TNF-α also can drive stem-cell like transition via EMT pathway in high-grade gliomas, leading to GSC-related tumor progression and therapy resistance.

GSCs are often resistant to conventional first-line treatments, such as radiation and chemo-therapies, due to the fact that stem-like cells in inflammatory TME can temporarily enter the quiescent G0 state in cell cycle, whereas conventional therapies only eradicate actively proliferating cells [55]. Additionally, GSCs have a high expression level of ATP binding cassette transporter G2 (ABCG2) that will potentially increase the chemotherapeutic drug efflux, diminishing treatment efficacy [56]. Immunotherapies also have poor efficacy on GSC elimination, partially because GSCs increase PD-L1 expression to exhaust T cells and secrete numerous chemokines as well as cytokines to induce M2 TAM polarization and accumulation of anti-inflammatory immune cells, such as Treg and MDSCs [57].

Therefore, PCD-induced inflammatory cell recruitment assists in GSC expansion. In turn, GSCs reinforce the immune suppression in TME while ensuring PCD to entice more immune cell infiltration and maintain the suppressive TME to transform incoming immune cells into anti-inflammatory pro-tumor subtypes. GSCs are the source for tumor adaptation under various treatment-induced hostile environment and eradicating GSCs is a critical target for innovative therapy model development (Fig. 2).

Overall, most anti-cancer modalities for GBM may result in PCD of tumor cells and an altered inflammatory TME, triggering tumor evolution and treatment resistance in a long-term exposure. Hence, it is of great importance to examine the few remaining highly effective treatment modalities for glioblastomas through the lens of PCD-tumor evolution theory.

### Lessons from treatment for GBM

#### Lessons from first-line standard treatment

Currently, the first-line standard management for GBM is the “Stupp’s Regimen” that includes radiation combined with concomitant and adjuvant temozolomide (TMZ) [58]. It has been reported that both radiotherapy and TMZ chemotherapy trigger PCD to eliminate tumor cells. As an alkylating agent, TMZ is able to methylate DNA at guanine residues to initiate mismatch repair as well as the resultant double strand breaks, and Ca^2+^-dependent caspase 3 overexpression with elevated Bax/Bcl-2 ratio, inducing apoptotic cell death [59–61]. In addition to apoptosis, autophagy is also activated upon radiation and TMZ in GBM cells and act to halt tumor growth via the degradation of oncogenic proteins, stimulation of AMPK and inhibition of Akt/mTOR signaling [59, 62, 63]. The application of autophagy inhibitor can abolish the tumor-killing effect of TMZ in in vitro experiments [64]. Moreover, radiotherapy and TMZ promote ferroptosis, the most common type of PCD in malignant gliomas, to eliminate GBM cells [22, 65, 66]. Some studies have discovered that ferroptosis can in turn improve TMZ’s cytotoxic activity [67, 68]. As proved by Hanjie Liu and colleagues, GBM cell lines that have acquired TMZ resistance are more susceptible to the ferroptosis inducer, erastin, than TMZ-sensitive GBM cells [69].

However, accumulating evidence indicates that various types of PCD contribute to TMZ resistance and tumor evolution. Autophagy serves a dual function, with both tumor-promoting and tumor-suppressing characteristics depending on the stages of tumorigenesis [59]. An analysis of expression data from 467 GBM samples reveals that GBM patients with higher expression of autophagy genes have worse prognosis and damage-regulated autophagy modulator (DRAM1) gene upregulation is linked to the activation of mitogen-activated protein kinase (MAPK) in GSCs, playing a role in TMZ resistance [70]. In preclinical models, DNA injuries induced by TMZ force glioma cells to increase ATP production as a stress response, protecting tumors from chemotherapy and maintaining the survival of GSCs [70, 71]. Apart from autophagy, several investigations have shown that ferroptosis can affect iron metabolism and enhance GSC growth, resulting in TMZ resistance [72]. In U87 and U251 GBM cell lines, although erastin can stall tumor proliferation, it also strengthens the migratory ability of tumor cells [69]. The conflict results in these recent researches, in our opinion, are due to the timing of PCD induction. In the early phase of tumor evolution, PCD can inhibit tumor cell proliferation and act synergistically with TMZ. Nevertheless, tumor progression is a chronic event, and enduring PCD of tumor cells will prompt tumor evolution as well as TMZ resistance. As a result, the PCD risk scores designed from tumor tissues of GBM patients and real-world GBM database are found to be inversely associated to patient survival time, contrary to the results from in vitro experiments [37, 69]. Other than directly assisting in tumor adaptation, PCD also sustains tumor growth and promote TMZ insensitivity by reshaping the TME into a highly immune suppressed environment. With machine learning, it is discovered that GBM patients who have little response to TMZ therapy are associated with higher expression levels of immune checkpoints (PD1, PD-L1, PD-L2) [73]. In TMZ resistant glioblastoma cell lines, expression levels of pyroptosis-related genes, genes associated with regulatory T cells and immune checkpoints are significantly higher than TMZ sensitive GBM cells [74]. These findings suggest that PCD and the long-term immune suppression in TME it induced might lead to TMZ resistance.

#### Lessons from immunotherapies

##### Immune checkpoint inhibitors (ICIs)

Great breakthrough in GBM immunotherapy has been observed over the previous decades, and ICIs have gained a lot of attention [75]. Well-known ICIs, such as pembrolizumab and nivolumab, have altered the clinical management regimen for a variety of malignancies, including NSCLC and melanoma [76, 77]. The promising results in other cancers pique our curiosity in the role of ICIs in GBMs. Given that chemotherapy-resistant GBM cells have typically evolved to be insensitive to the intrinsic apoptotic pathway triggered by TMZ-induced DNA damage, the mechanism by which ICIs eradicate cancers involves activating extrinsic cell death pathway via enhancing the release of death signals from surveilling immune cells, as shown in preclinical studies [78, 79]. In addition, CD47-targeting immune checkpoint inhibition can induce GBM cell death via Akt/mTOR inactivation and elevated autophagic flux in glioblastoma cell lines [79, 80]. Nonetheless, ICIs failed to demonstrate efficacy in the clinical studies. The Keynote-028 clinical trial explores pembrolizumab monotherapy in 26 recurrent GBM patients and finds that it provides only marginal survival advantages [81]. Furthermore, the results of the first large-scale phase 3 clinical study investigating the effectiveness of ICIs for GBM are likewise unsatisfactory, with nivolumab monotherapy failing to extend survival time when compared to the bevacizumab-treated control group [82]. Afterwards, researchers try to combine ICIs with first-line therapies in pGBM (Checkmate 498 and Checkmate 548), but neither trial demonstrates significant survival improvement [83, 84]. Currently, ICI application before surgery can increase chemokine release, immune cell infiltration, and clonal diversity among tumor-infiltrating T lymphocytes, indicating a bright future for neoadjuvant immunotherapies in GBMs [85]. Interestingly, the scientists observe that neoadjuvant PD-1 checkpoint blockade can successfully enhance the infiltration and clonocal expansion of T cells and conventional type 1 dendritic cells (cDC1s), but it cannot overcome the immunosuppressive TAMs in recurrent GBM [86]. The difficulty is primarily caused by the co-evolution of tumor cells and TME. ICIs may activate T cells to secrete numerous pro-inflammation cytokines to eliminate malignant tumor cells, but at the same time ICIs may stimulate the production of immunosuppressive molecules such as PD-L1, IDO1, and IL4I1, resulting in M2 macrophage polarization and T-cell activity inhibition [86]. Therefore, future studies should target TAMs to complement the efficacy of neoadjuvant ICIs.

##### Adoptive immunotherapy

Chimeric antigen receptor (CAR) T-cell therapy is a promising new therapeutic option for GBM. CAR-T cells are created from genetically modifying T cells armed with CARs targeting particular antigens on GBM cells, such as EGFRvIII, IL13Ra2, and B7-H3, ushering in a new age of individualized cancer therapy [87]. Despite some encouraging results, each CAR-T therapy only aims at a single tumor antigen, imposing the survival pressure on tumor cells. Overtime, GBM cells will evolve by partial or total loss of target antigen expression, leading to therapeutic resistance, a phenomenon known as antigen escape. For example, in 2017, the first clinical trial (NCT02209376) of CAR-T EGFRvIII in 10 recurrent GBM patients with EGFRvIII-positive tumors is conducted and five of the seven patients who received post-CAR-T reoperation has reduced EGFRvIII in resected tumor tissues [88]. Based on this phenomenon, ongoing clinical investigations focus on the development of CAR-T therapies that target multiple tumor antigens.

#### The prospects of relatively indiscriminate treatments

It is true that the induction of PCD and inflammatory TME can hinder tumor proliferation, but we should consider treatment efficacy in terms of tumor evolution. Specifically, the intensity and duration of therapy are crucial since they are closely associated with tumor adaptation and treatment resistance. Due to the selectivity of blood brain barrier (BBB), many chemotherapies and immunotherapies cannot reach adequate concentration in CNS and BBB also hampers immune cell infiltration. As a consequence, these treatments are not concentrated enough to kill tumor cells and will instead reshape the TME to which tumor cells can slowly adapt under the selective pressure. Therefore, it is necessary to combine numerous physically indistinguishable therapeutic modalities, like Tumor-treating fields (TTFields), hypofractionated radiotherapy (HFRT) and laser interstitial thermotherapy (LITT) to improve effectiveness and prevent tumor progression (Fig. 2). These localized physical treatments exert a potent tumor cell destruction effect and suppress tumor evolution due to their indiscriminate killing mechanisms, unlike the targeted killing actions of targeted therapies. Table 1 summarizes therapies with promising prospects and the multi-modal combinations that are currently under clinical investigation.

**Table 1:** Clinical trials of relatively indiscriminate treatments for GBM. Combination treatments amplifies the effect for GBM

Treatment	Combination	Design	Outcome (PFS, OS)	Phase	References
**TTFields**	pulsed Bev	rGBM: pulsed Bev (one cycle on, one cycle off (8w/cycle)	mOS 7.4 months (terminated insufficient n)	Phase 2	NCT02663271
**TTFields**	Bev	rGBM: Bev + TTF	mOS 10.5 months mPFS 4.1 months	Phase 2	NCT01894061
**TTFields**	Bev + RT	Bev-naïve rGBM Bev + TTF for 4wk RT start after 3 cycles of Bev; hypofractionated RT adjuvant Bev + TTF	terminated (low accrual)	pilot study	NCT01925573
**TTFields**	Bev + TMZ	nGBM RT/TMZ + Bev = > after RT: TTF + TMZ + Bev	terminated (low accrual) mOS 9.9 months mPFS 7.9 months	Phase 2	NCT02343549
**TTFields**	chemoradiation	TMZ + RT	ongoing	Phase 2	NCT04902586
**TTFields**	SRS	7d = > SRS on MRI or FET-PET (5d) => restart TTF	ongoing	Phase 2	NCT04671459
**TTFields**	Pembrolizumab	TMZ/RT = > TMZ & TTF & Pembrolizumab	mOS 25.2 months (control: 15.9 months) mPFS 12.1 months (control: 7.9 months) case-matched control	Phase 2	NCT03405792
**TTFields**	Niraparib	Without surgery: niraparib + TTF Surgery: TTF = > surgery = > TTF + niraparib	ongoing	Phase 2	NCT04221503
**TTFields**	TMZ, RT	TMZ/RT = > TTF + RT + TMZ (RT: 30 fractions, 5d/w)	ongoing	pilot study	NCT03477110
**TTFields**	mutation-derived tumor antigen vaccine (MTA-based vaccine)	MTA-based personalized vaccine (peptides + poly-ICLC) + TTF	ongoing	Phase 1	NCT03223103
**TTFields**	TMZ, RT	TTF + TMZ + 5d hypofractionated RT (35 Gy in 5d from day2)	ongoing	Phase 1	NCT04474353
**TTFields**	RT, TMZ, chloroquine	3D CRT or IMRT + TMZ & chloroquine for 49d adjuvant: 4wk after RT, TMZ + chloroquine	ongoing	Phase 1	NCT04397679
**TTFields**	concomitant RT/TMZ (EF-32)	nGBM arm I: concurrent TMZ/RT/TTF + adjuvant TMZ/TTF arm II: TMZ/RT + adjuvant TMZ/TTF	ongoing	RCT	NCT04471844
**TTFields**	nivolumab, ipilimumab	rGBM arm I: Nivo + TTF arm II: Nivo + Ipli + TTF	end enrollment early	Phase 2	NCT03430791
**HFRT**	Avelumab	Avelumab + HFRT (30 Gy/5fx)	mOS 10.1 months mPFS 4.2 months	Phase 2	NCT02968940
**HFRT**	Bevacizumab + TMZ	HFRT + TMZ + Bevacizumab	mOS 8.5	Phase 2	NCT01478321
**HFRT**	Hyperbaric oxygen therapy	Hyperbaric oxygen therapy + HFRT (5 Gy for 3–5 times)	mOS 10.7 months mPFS 5.2 months	Not Applicable	NCT03411408
**HFRT**	Bevacizumab	Group A: HFRT Group B: HFRT + Bevacizumab	Group A mPFS 7.6 months mOS 12.1 months Group B mPFS 4.8 months mOS 12.2 months	Phase 2	NCT01443676
**HFRT**	TMZ	Group A: HFRT ( 40 Gy/15fx) + TMZ Group B: HFRT ( 40 Gy/15fx)	Group A mOS 9.3 months mPFS 5.3 months Group B mOS 7.6 months mPFS 3.9 months	Phase 3	NCT00482677
**LITT**	TMZ + radiotherapy	IDH wild-type WHO grade 4 GBM	nGBM Chemo + radiation by 12 weeks: mOS 16.14 mPFS 11.93; nGBM Chemo/radiation alone, or neither at 12 weeks: mOS 5.36 mPFS 3.88	—	NCT02392078
**LITT**		nGBM LITT = > Concurrent chemoradiation begin within 7d	ongoing	Phase 1	NCT02970448
**LITT**		Control group: biopsy + adjuvant treatment Experimental: Biopsy + LITT + adjuvant treatment	ongoing	Phase 3 RCT	NCT05318612
**LITT**	Pembrolizumab	Pembrolizumab given every 3 weeks starting no more than 1 week after LITT until progression or unacceptable toxicity	patient1: OS 40 PFS 33 patient2: OS 12 PFS 12 patient3: OS NR PFS 7	Phase 1 & 2	NCT02311582
**LITT**		Pembrolizumab injections 7 days before = > LITT = > Pembrolizumab at 14 days post = > Pembrolizumab at 35 days post	ongoing	Phase 1 & 2	NCT03277638
**LITT**	Avelumab	Part A - Avelumab administered intravenously every 2 weeks 10 mg/kg for 2 cycles Part B - Avelumab + MRI-guided LITT therapy	completed but no result	Phase 1	NCT03341806
**LITT**	Doxorubicin, Etoposide	arm A: LITT => DCE and DSC-MRI imaging Arm B: LITT = > doxorubicin IV for 6w = > etoposide PO 21 days of each 28-day cycle = > DCE and DSC-MRI imaging	ongoing	Phase 2	NCT02372409
**LITT**	Doxorubicin	Arm B: LITT = > 6-8w later doxorubicin hydrochloride IV 20 mg/m2 over 5 min once weekly for 6 weeks = > Biomarker blood draws = > DSC-MRI Arm C: LITT = > within 72 h later doxorubicin hydrochloride IV 20 mg/m2 IV over 5 min once weekly for 6 weeks = > DSC-MRI	completed but no result	Phase 1	NCT01851733
**LITT**	Hypofractionated Radiation Therapy	LITT = > hypofractionated RT (once daily on consecutive days, within 10 days of the LITT treatment)	ongoing	Not Applicable	NCT04699773 NCT04181684
**LITT**	F18 Fluciclovine	estimate accuracy of F18 Fluciclovine PET MR for LITT	ongoing	early phase 1	NCT05054400
**LITT**	Lomustine	LITT = > receive Lomustine PO on day 1 = > Lomustine repeats every 42 days for up to 6 cycles	terminated	Phase 2	NCT03022578
**LITT**	—	(i) biopsy and LITT (ii) biopsy alone	completed but no result	Randomized, Pilot	NCT04596930
**LITT**		TRANBERG Thermal Therapy System and TRANBERG Thermoguide Workstation.	ongoing	Not Applicable	NCT05296122
**LITT**		Auto LITT system	completed but no result	Phase 1	NCT00747253

##### Tumor-treating fields (TTFields)

As a novel anti-tumor treatment, TTFields inhibits cell division via alternating electric fields of intermediate frequency (∼100–500 kHz) and low intensity (1–3 V/cm) [89]. TTFields not only increases the BBB permeability, but also enhance various anti-tumor signaling pathways, such as anti-tumor immune response, anti-mitotic signaling, and DNA damage repair pathway [90]. When treating patient-derived glioma stem-like cells with TTFields, TTFields therapy disrupts DNA damage repair system and the functioning of replication fork, significantly increasing level of apoptotic cell death in previously treatment-resistant GSCs [91]. Apart from apoptosis, studies demonstrate that TTFields-treated GBM cells have elevated autophagic flux, stimulating ATP production that function as a signal to attract immune cells [92, 93]. In addition to its direct inhibitory effects on GBM cells, TTFields alters the TME of GBM cells, particularly the immunological TME through pyroptosis, to boost antitumor immune response, indirectly limiting tumor growth [20]. In murine lung and colon cancer models, it is verified that TTFields cause immunogenic cell death via pyroptosis and can improve antitumor efficacy when combined with anti-PD-1 therapy [92]. For GBM, ongoing phase II clinical trial(NCT03405792), the mOS in combination of TTFields, TMZ and anti-PD-1 therapy group is 24.8 months [94]. Preclinical studies demonstrate that TTFields can increase chemotherapy sensitivity in human glioblastoma cell lines and animal models, providing the theoretical basis for combination therapies with other GBM treatment (Table 1). TTFields as an add-on to traditional radiotherapy and chemotherapy has emerged as a breakthrough in the clinical management of GBM [95]. In a phase 3 clinical trial, adding TTFields to maintenance temozolomide chemotherapy significantly prolonged progression-free and overall survival in GBM patients [96]. Preclinical research supports the use of TTFields treatment immediately following radiation therapy (RT) as a feasible regimen for improving RT outcome [97]. TTFields combined with targeted therapy, such as bevacizumab, an inhibitor of VEGF, and dabrafenib, an inhibitor of BRAFV600E, can prolong survival [98]. Additionally, in preclinical research, it is reported that TTFields can downregulate stem cell markers, and promote the efficacy of proton beam [99]. All of this evidence has shown that TTFields has enormous potential in GBM treatment.

##### Hypofractionated radiotherapy (HFRT)

Hypofractionated radiation therapy (HFRT) offers several advantages. HFRT is now widely accepted for treating patients with poor physical conditions. HFRT is preferred and recommended RT modality for patients with astrocytoma, oligodendroglioma, or glioblastoma who have a Karnofsky performance status (KPS) score less than 60, according to the 2022 National Comprehensive Cancer Network (NCCN) recommendations [100]. Additionally, due to the safety and tolerability of HFRT, even senior glioblastoma patients in good physical condition might consider it as a therapy option [101]. HFRT has improved tumor-killing capacity by giving a larger radiation dosage each time and shorten the overall treatment course [102, 103]. Radiation has been proven in vivo and in vitro to promote glioma cell death by oxidative stress, DNA damage, and apoptosis [104]. However, in vitro studies suggest that irradiation at lower doses, despite capable of initiating autophagy initially, failed to kill cancer stem cells and the increased autophagic flux provide energy as well as metabolic building blocks for GSCs, thus leading to GSC proliferation and tumor evolution [105, 106]. On the contrary, HFRT deliver high doses of radiation during each session, eliminating glioma stem cells and preventing tumor resistance [107, 108]. In addition, HFRT drastically shortens the treatment time, while conventional radiation (cRT) takes longer. Patients in poorer health state may drop out midway through treatment process, and tumor cells may re-grow during treatment [109]. The goal of HFRT is to complete the entire therapy process in three weeks [110]. Clinical evidence suggests that HFRT may slow the pace of GBM cell repopulation and improve patient compliance [111]. Following HFRT, the transcriptomes of glioma stem cells also change dramatically, perhaps contributing to the improved long-term clinical outcomes [112]. Lastly, HFRT may modulate GBM microenvironment to boost the immune response, thereby improving the effectiveness of immunotherapy [113]. At low doses of radiotherapy delivered by conventional radiotherapy, GBM TME exhibits a buildup of radioresistant immunosuppressive cells, including M2 type of tumor-associated macrophages, myeloid-derived suppressor cells and regulatory T cells [114–116]. However, high dosages of radiation as in HFRT shift suppressive TME into a supportive one, with increased infiltration of pro-inflammatory immune cells and APCs [114, 117]. Hence, anti-PD1 antibodies, when combined with HFRT, appear to provide a longer lasting anti-tumor action, potentially doubling survival time [118]. These findings highlight that HFRT as a treatment modality can modulate TME and hinder tumor evolution of malignant gliomas.

##### Laser interstitial thermotherapy (LITT)

As a minimally invasive technique, LITT has gained a lot of attention in treating CNS malignant tumors [119]. LITT works by introducing an optical fiber into the tumor under MRI navigation followed by laser heating tumors to increase local hyperthermia and anticancer activity [119]. Currently, intraoperative MR thermometry can achieve precision tumor targeting and accurately deliver therapeutic heat doses with real-time observation of tissue damage [120]. LITT, as a physical treatment mode, has been used to treat epilepsy, metastatic brain cancers, and gliomas for decades [121]. In glioblastoma, LITT has equivalent efficacy as surgical resection. In a multicenter prospective study of LITT in IDH wild-type glioblastoma, de Groot JF et al. find that patients with GBM treated with LITT and postoperative chemoradiotherapy has an OS of 16.14 months and a PFS of 11.93 months, comparable to conventional surgical resection, indicating that LITT can be a good option for those not suitable for surgery [122]. At ablation temperature near the tip of laser, the heat trigger mitochondria damage and dysfunctional DNA repair, activating intrinsic pathway of apoptosis [123–125]. In addition to the direct killing of tumor cells through thermal effects, LITT also acts synergistically with chemotherapy and immunotherapy by increasing BBB permeability and inducing immunogenic cell death, while preventing tumor evolution through the timely elimination of residual GBM cells [126]. At a further distance from the laser tip, tumor tissues are exposed to hyperthermia, a temperature range that does not induce apoptosis but rather triggers immune activation [125]. Hyperthermia can enhance antigen presentation by APCs, partially because of accelerated maturation of dendritic cells, and facilitate activation as well as migration of T cells [125, 127]. The current ongoing clinical trials involving LITT in GBM are summarized in Table 1.

## Conclusion

Glioblastoma is a subtype of glioma characterized by a dismal prognosis and limited treatment options. Because high-grade gliomas have extraordinary plasticity, these tumor cells can evolve to avoid destruction imposed by anti-tumor therapies. Inflammatory TME, EMT signaling, hypoxia, and angiogenesis all contribute to survival pressure, which in turn aids tumor evolution. This review aims to address the controversies regarding the efficacy of PCD-based therapies. Short-term and intense PCD can indeed cause tumor cell death by activating apoptosis, autophagy, ferroptosis pyroptosis and other forms of PCD. However, due to the constraints imposed by the permeability of blood brain barrier, long-term systemic administration of PCD-based therapeutics often immerse GBM cells in sub-optimal concentrations. As a result, chronic and inadequate PCD eventually leads to epithelial-mesenchymal transformation and accumulation of GSCs, thereby promoting tumor evolution and treatment resistance. Targeted treatment modalities, including temozolomide and immunotherapies, have limited effectiveness in GBM due to tumor adaptation upon PCD induction. In contrast, indiscriminate therapeutic methods such as TTFields, HFRT and LITT can deliver intensified stimulation of PCD while simultaneously halting the EMT transformation of GBM cells. Therefore, these indiscriminate therapies not only induce strong and localized PCD-related tumor cell death to prevent tumor evolution, but also inhibit the formation of glioma stem cells, averting tumor progression. The combination of these indiscriminate therapies with other regimens is expected to be the future trend in GBM management.

## Data Availability

No datasets were generated or analysed during the current study.

## Abbreviations

ABCG2: ATP binding cassette transporter G2
APCs: antigen-presenting cells
Bev: Bevacizumab
CAR: Chimeric antigen receptor
CNS: Central nervous system
GBM: Glioblastoma
HFRT: Hypofractionated radiotherapy
ICIs: Immune checkpoint inhibitors
LGG: low-grade glioma
LITT: Laser interstitial thermotherapy
NES: Natural evolution signature
PCD: Programmed cell death
TME: Tumor microenvironment
TMZ: Temozolomide
TTFields: Tumor-treating fields
NCCN: National Comprehensive Cancer Network
